# A Multifaceted Resident-Led Physical Exam Course

**DOI:** 10.15694/mep.2021.000109.1

**Published:** 2021-05-03

**Authors:** Ryan Dean, Dustin Morrow, Steven Connelly

**Affiliations:** 1 Prisma Health-Upstate

**Keywords:** medical education, physical exam, ultrasound, resident as teachers, medical decision making

## Abstract

This article was migrated. The article was marked as recommended.

Background:

Physicians frequently report poor confidence applying the physical exam for medical decision making. We developed a novel, multifaceted, resident-led curriculum to teach medical students the physical exam for clinical practice.

Methods:

We created a two-week elective comprised of didactics, journal discussions, bedside ultrasound, and physical exam rounds for fourth year medical students at the University of South Carolina School of Medicine - Greenville. *JAMA: The Rational Clinical Exam* and *Evidence-Based Physical Diagnoses,* by Steven McGee, MD, were used to develop content. The curriculum focused on cardiac, pulmonary, abdominal, endocrine, and neurologic exams. Faculty and residents facilitated all portions of the course. Chi-squared testing was used to calculate confidence intervals on pre- and post-course assessments.

Results:

Twenty-two fourth year medical students enrolled in the elective over the course of three years. Seventeen faculty, three chief residents, and 13 residents provided instruction. Residents provided roughly half of the total instruction hours. Students demonstrated statistically significant improvement on multiple choice pre-course and post-course assessments (56.8% vs 77.1%, p < 0.001). 95.5% of students reported feeling “confident” in their physical exam skills after the course.

Conclusion:

After participating in the course, students demonstrated improved skill and comfort using the physical exam for clinical decision making.

## Introduction

Historically, the physical exam has been the cornerstone of clinical evaluation. Even with advancements in medicine and technology, the physical exam can still have a large impact on patient care ( Reilly, 2003; Verghese *et al.,* 2015) Despite the benefits of the physical exam, many physicians report poor self-confidence or demonstrable deficiencies in physical exam skills ( Paauw *et al.,* 1995; Mangione, 1997; Wu *et al.,* 2007). There are many possible explanations for such incompetence, including rote memorization of exam maneuvers during medical school training. Students subsequently have difficulty translating their knowledge to direct patient care ( Wilkerson and Lee, 2003; Allen *et al.,* 2017). In recent years, there has been a growing body of research supporting the use of evidence-based medicine, hypothesis driven exam techniques, and clinical reasoning to teach the physical exam during both preclinical and clinical years of medical school ( Fagan *et al.,* 2003; Alexander, 2008; Naghshineh *et al.,* 2008; Jasani and Saks, 2013; Perrig *et al.,* 2016; Allen *et al.,* 2017; Maguire, 2017; Wijeratne *et al.,* 2018). New literature has also demonstrated the utility of bedside point of care ultrasound (POCUS) to augment the accuracy of the physical exam ( Kobal *et al.,* 2005; Bouhemad *et al.,* 2007; Stokke *et al.,* 2014; Kimura, 2017).

We developed a novel, multifaceted physical exam course for fourth year medical students incorporating evidence-based medicine, physical exam rounds, simulation medicine, POCUS, and art appreciation with the help of resident and faculty volunteers. We hypothesized that medical student skill, knowledge, and comfort applying physical exam techniques would improve after completing the course.

## Methods

### Course description

We developed a two-week elective for fourth year medical students at the University of South Carolina School of Medicine - Greenville in collaboration with Greenville Health System in Greenville, SC. The elective underwent curriculum committee review and received approval with exemption from the local institutional review board (IRB). We compiled material for the course using articles from the *JAMA: The Rational Clinical Examination* (RCE) ( Simel and Rennie, 2009) and the *Evidence-Based Physical Diagnoses* text by Steven McGee, MD ( McGee, 2012). The medical school offered the elective from 2017 to 2019.

The course format incorporated the cardiovascular, pulmonary, gastrointestinal, endocrine, and neurologic organ systems. Independent readings, student-led article review, and interactive didactic sessions were devoted to each organ system. Materials focused primarily on the evidence behind exam maneuvers and proper technique. The schedule included additional lectures on biostatistics, cognitive biases, and art appreciation to develop “visual literacy” as time allowed.

Each afternoon, students rounded in the Greenville Memorial Hospital on patients with relevant physical exam findings pertinent to the organ system of study. Residents and faculty identified appropriate patients in advance and obtained verbal consent to participate in the activity. Students split up between volunteer residents and faculty to examine patients with a goal ratio of 3:1. Students practiced exam maneuvers and interpretation of the findings while facilitators discussed how findings might impact medical decision making. No patient health information was collected as a part of this study.

Students spent two days with POCUS trained emergency medicine and hospital medicine faculty in our Simulation Center. Each four-hour session utilized didactics and standardized patients to learn basic ultrasound orientation and image acquisition. Students also practiced scanning each other during unstructured time. Each afternoon, students applied their skills to patient care during bedside rounds in the emergency department and hospital.

In response to student feedback requesting more interactive sessions, we added two additional observational activities in 2018 and 2019. With the help of the Simulation Center, we developed an observational activity to identify medical error and waste based on a concept developed at the University of Chicago Medical Center ( Maguire, 2017). Students also participated in a rapid response simulated case of a patient with acute dyspnea.

### Assessments

We created multiple-choice pre-course and post-course assessments based on the planned content for the course. Objective structured clinical examination (OSCE) final assessments were also developed with standardized patients. During the encounters, standardized patients provided cards with physical exam findings or played audio of auscultatory findings in response to appropriate maneuvers and technique. Students submitted a write-up where they provided an assessment and plan based on the findings they uncovered. Due to time constraints, POCUS image acquisition was not included as part of the OSCE though representative images of POCUS findings were provided to students upon request. Each case lasted 30 minutes. Simulation Center staff trained standardized patients. We reviewed OSCEs and graded them based on history, maneuver choice, technique, assessment, and follow-up plan. The four cases used included 1) differentiating congestive heart failure exacerbation from chronic obstructive pulmonary disease exacerbation in a patient with shortness of breath; 2) identifying cirrhosis and spontaneous bacterial peritonitis in a patient with alcoholism and seizures; 3) identifying a cardiac etiology for syncope; and 4) identifying pneumonia in a patient with abdominal pain. In 2017 students performed all four cases. In 2018 and 2019 students performed only the first two cases due to time constraints.

Based on feedback from students in 2017, we created an additional multimedia quiz in 2018 to better assess features of the physical exam that were not previously tested. The quiz incorporated sound files, pictures, and video clips into short clinical vignettes with multiple choice or short answer questions.

We asked students to rate each session using a five-point Likert scale grade and an open response. At the end of the elective students sat down with the authors and provided feedback for the course.

### Grading

The medical school offered the two-week elective as a pass/fail course for students. We tabulated grades based on composite scores from the post-course assessment, oral exam, and the multimedia assessment (2018 and 2019 only).

### Statistical analysis

Our biostatisticians aggregated student demographic data and performance. They compared the change in scores from the pre-course assessment and post-course assessment using a Chi-squared technique to determine statistical significance with a 95% confidence interval.

## Results/Analysis

### Demographics

**Figure 1:**
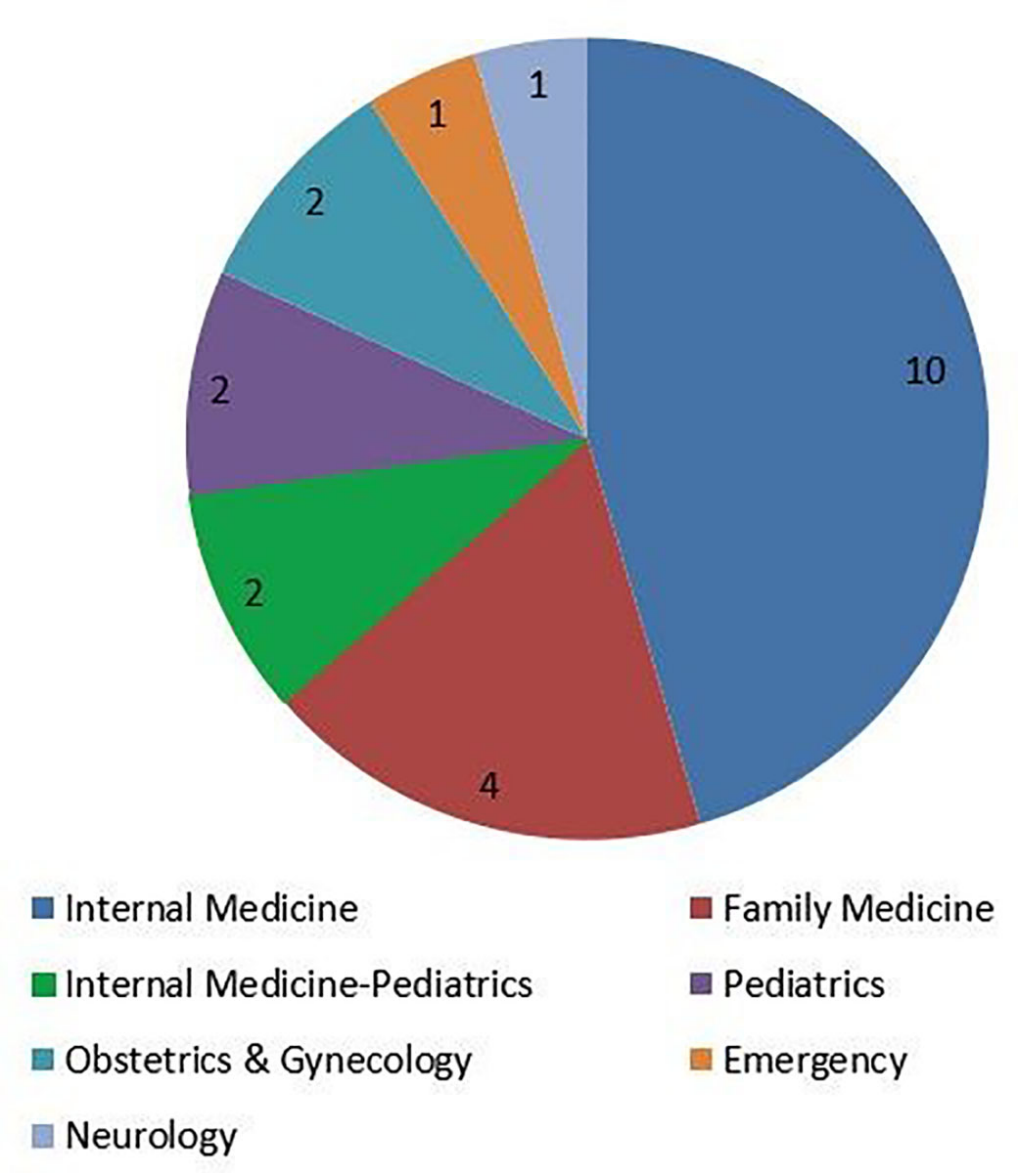
Number of medical students enrolled by intended residency specialty.

A total of 22 students took the course over a three-year timespan. Ten students planned to match into internal medicine (45.5%) and four into family medicine (18.2%). The remaining students anticipated matching into internal medicine-pediatrics, pediatrics, obstetrics-gynecology, emergency medicine, and neurology ( Figure 1). Sixteen women and six men enrolled in the course.

### Volunteers

A total of 17 faculty, 3 chief resident, and 13 residents helped facilitate the course. Residents contributed roughly half of the total time teaching. Residents were primarily in their post-graduate year (PGY) three or four, though one was a PGY-2.

### Assessments

**Figure 2:**
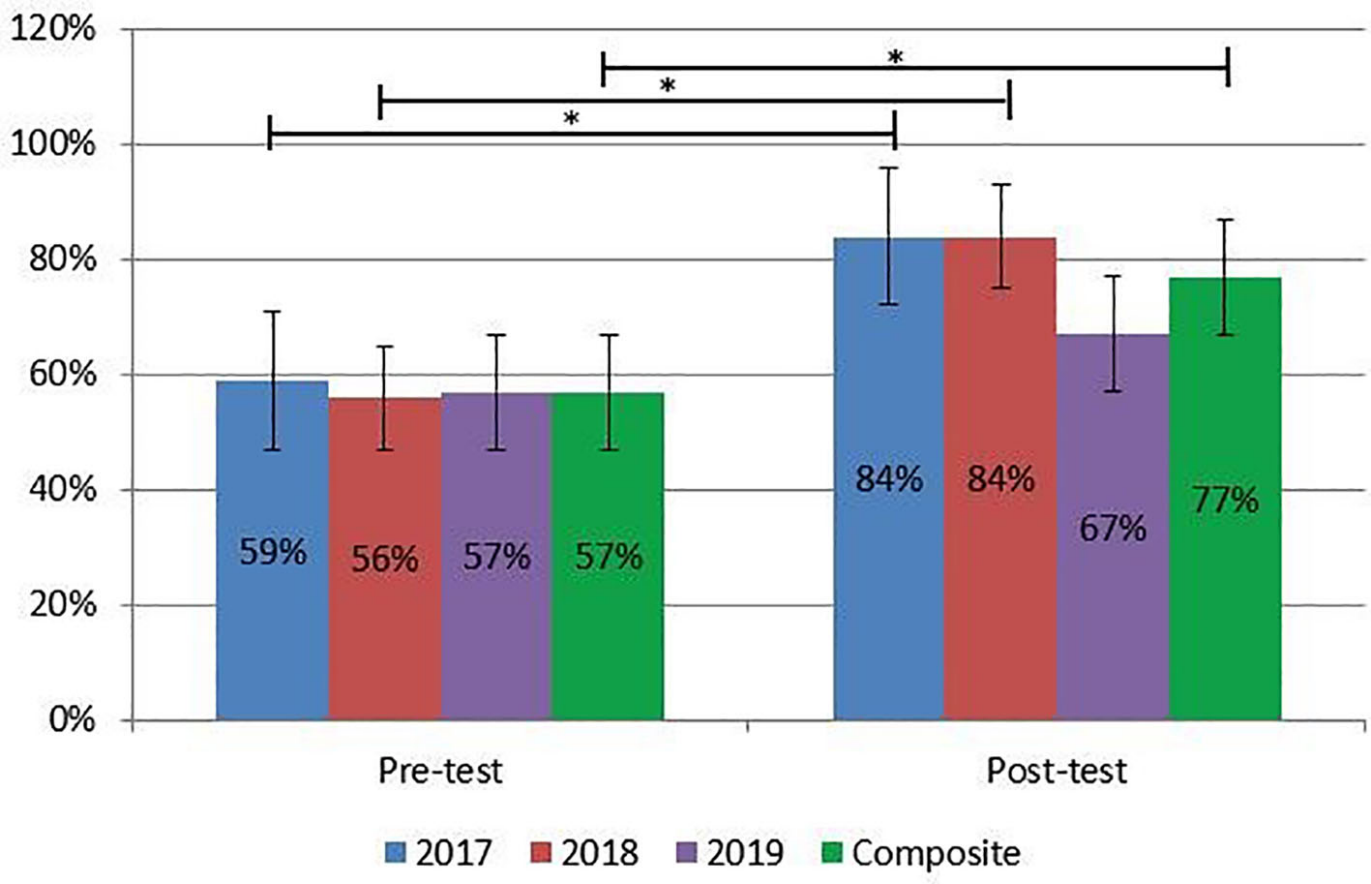
Multiple choice assessment results by year. * signifies statistical significance (CI 95%).

Students scored an average of 12.4/21 points (59.1 ± 12.9%); 11.1/20 points (55.6 ± 11.5%); and 11.3/20 points (56.7 ± 10.6%) on their pre-course assessments in 2017, 2018, and 2019 respectively ( Table 1 and Figure 2). Overall students correctly answered on average 56.8 ± 11.0% of all pre-course assessment questions. After completion of the course, students earned 16.8/20 points (84.0 ± 11.4%), 16.9/20 points (84.4 ± 6.8%), and 13.3/20 (66.7 ± 9.4%) in 2017, 2018, and 2019, respectively. Students correctly answered on average 77.1 ± 12.3% of all post-course assessment questions. Students demonstrated a statistically significant improvement in scores from 2017, 2018, and composite scores. Twenty of 22 students improved their scores while one score remained the same and one worsened.

**Table 1:** Multiple choice assessment results by year.

Year	N	% Pre-Course Score ^a^	% Post-Course Score ^a^	p-value
2017	5	59.05 ± 12.87	84.00 ± 11.40	0.006
2018	8	55.63 ± 11.48	84.38 ± 6.78	< 0.001
2019	9	56.67 ± 10.61	66.67 ± 9.35	0.05
Overall	22	56.83 ± 10.96	77.05 ± 12.31	< 0.001

^a^ percent mean ± standard deviation

**Figure 3:**
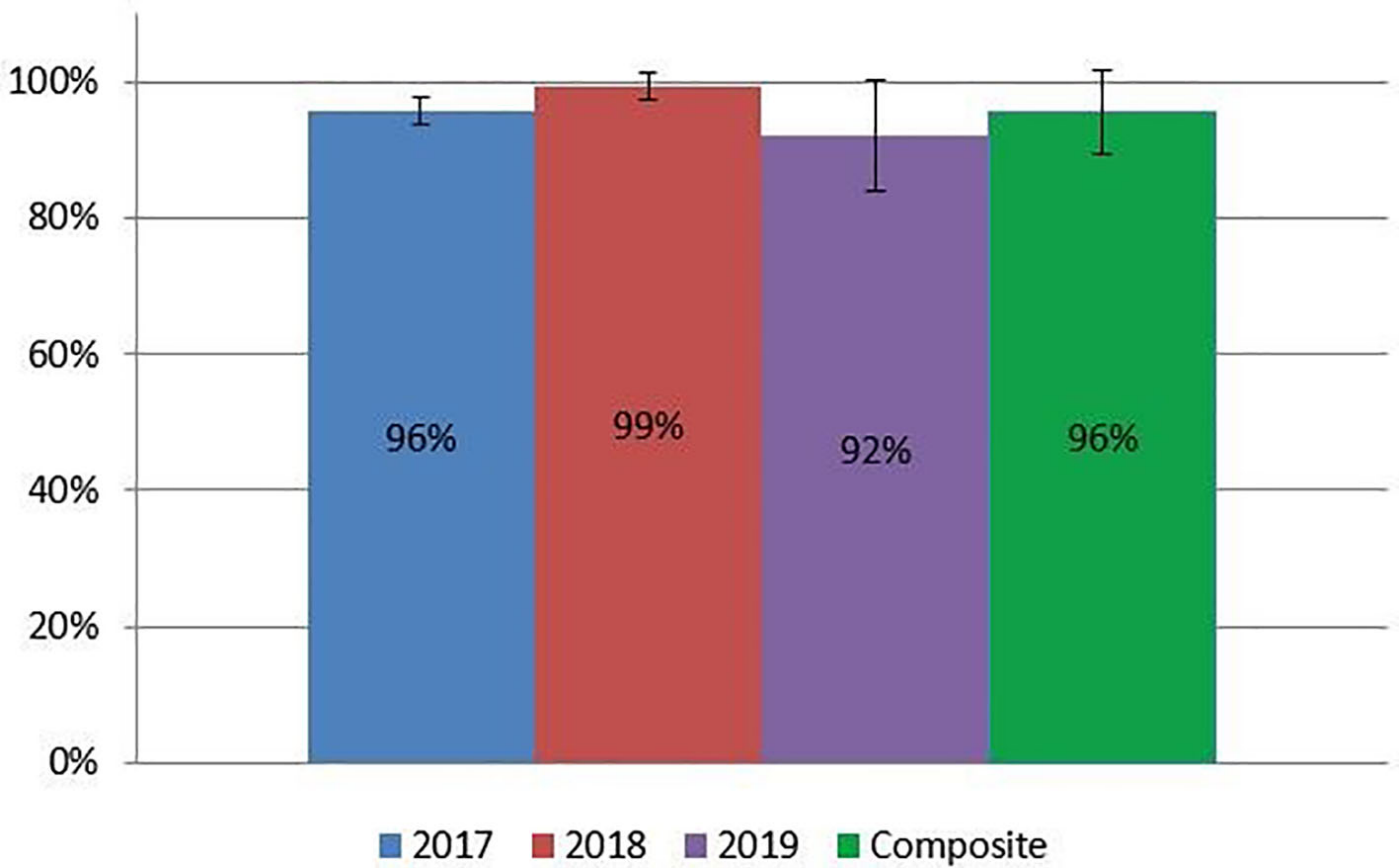
OSCE assessment results by year.

**Figure 4:**
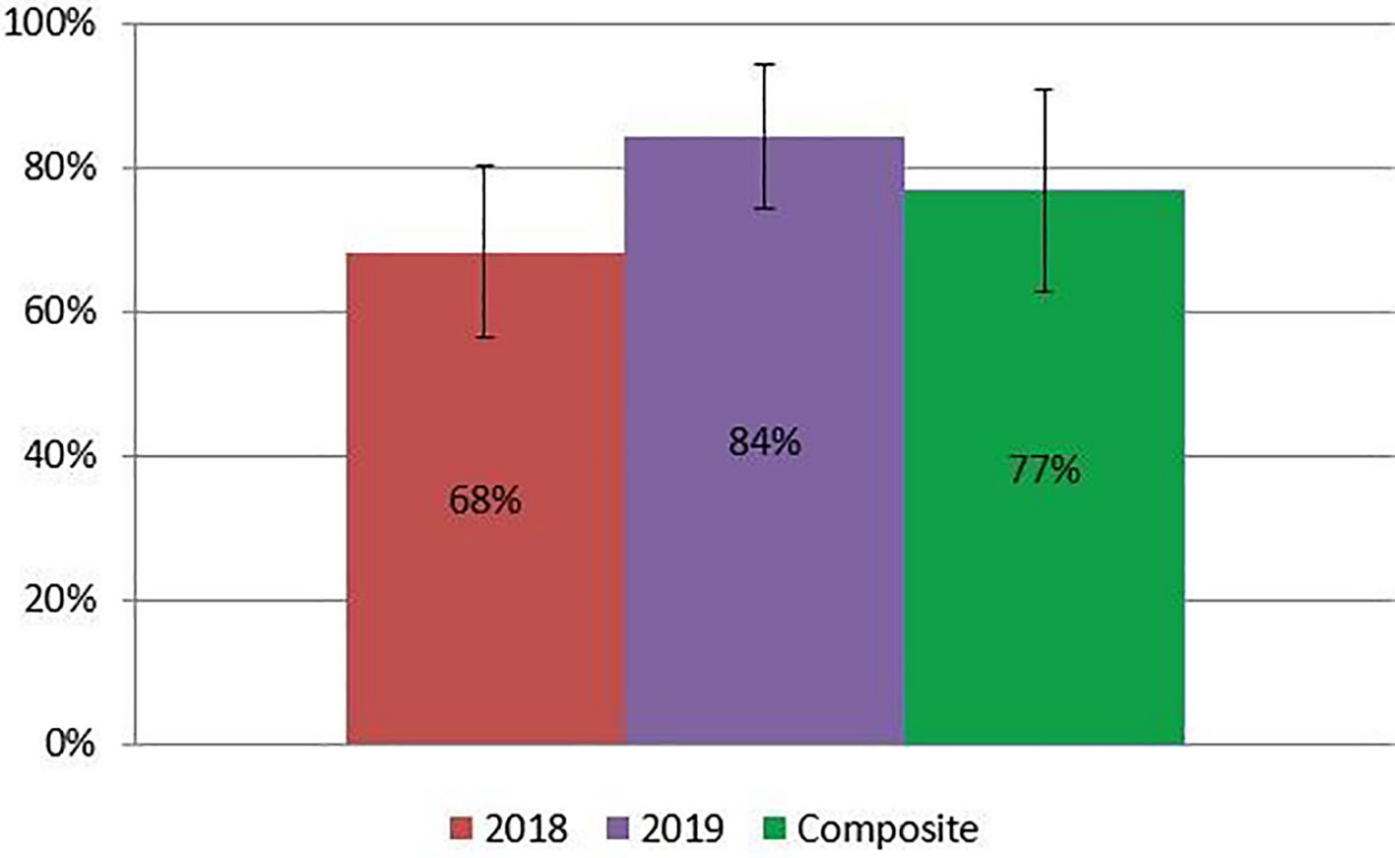
Multimedia assessment results by year.

On final OSCE assessments, students earned 43.0/45 points (95.6 ± 2.2%) in 2017; 29.8/30 points (99.2 ± 2.4%) in 2018; and 27.6/30 (92.1 ± 7.8%) in 2019. In 2017, we excluded case three from grading due to technical difficulties preventing completion of the grading rubrics. Collectively, participants scored 95.5 ± 5.7% on their OSCE assessment ( Figure 3). The multimedia assessment introduced in 2018 produced an average score of 10.3/15 points (68.3 ± 12.2%) in 2018; 12.7/15 points (84.4 ± 10.4%) in 2019; and 76.9 ± 14.0% between both years (Figure 4). Finally, students earned an average final grade of 59.8/65 points (92.0 ± 3.3%) in 2017; 56.9/65 points (87.5 ± 5.2%) in 2018; 53.7/65 points (82.6 ± 5.7%) in 2019; and 86.5 ± 6.1% overall. All students passed the course according to academic standards set at the medical school.

### Feedback

In follow-up surveys, students reported the article discussions, bedside exam rounds, and ultrasound trainings were most helpful for learning the physical exam. They felt the didactic sessions were not sufficiently interactive. Despite training, some of the standardized patients had difficulty providing physical exam cards during the OSCEs. The students thought the multimedia assessment accurately tested content from the course. When asked “After taking this class do you feel more confident in your physical exam skills and medical decision making?”, 21 out of 22 students reported they were “confident” or “very confident.” Similarly, 21 out of 22 students reported they were “very likely” to use the concepts taught in the course for further practice.

## Discussion

Over the course of the two-week elective, students demonstrated an improvement in their knowledge as evidenced by the improved pre- to post-course assessments. Results from the OSCE and multimedia assessment also demonstrated students were able to apply their knowledge of the physical exam clinical scenarios, though there was no pre-course comparison. While scores remained similar between the 2017 and 2018 cohorts there was a slight drop in average score across assessments in 2019. Multiple reasons may explain this difference, including the addition of an extra grader, different teachers, and differences in the quality of findings for exam rounds. Additionally, there were more excused absences resulting in missed didactics and exam rounds in 2019. Of the three assessments, students felt the multimedia assessment best tested their knowledge acquired during the course.

Investigators have developed many different types of physical exam courses across the country. for instance, Allen *et al.* developed a course introducing the hypothesis driven exam during preclinical years of training ( Allen *et al.,* 2017). Faculty also studied the impact of innovative curricula and observational activities on visual awareness ( Naghshineh *et al.,* 2008; Jasani and Saks, 2013; Maguire, 2017). Many schools incorporate various components of evidence-based medicine, visual literacy, and clinical decision-making to their physical exam course, but few have sought a comprehensive curriculum ( Uchida *et al.,* 2019). Fagan *et al.* (2003) showed a significant improvement in knowledge of and skill with the physical exam after completion of an 8-week multifaceted curriculum comprise for article review, didactic, and practice with real patients. Similarly, our course demonstrated improvement over the course of two weeks using a multi-faceted approach to teach the physical exam. Additionally, multiple studies have reported medical student ability to apply POCUS after training with a high degree of precision and accuracy ( Kobal *et al.,* 2005; Stokke *et al.,* 2014). While our course did not directly measure a change in POCUS application, our students did demonstrate competency interpreting ultrasound imaging on our post-course assessments.

One unique feature of our course was the large resident involvement. Residents provided roughly half the hours volunteered and much of the curriculum development. This commitment took some of the burden of teaching off faculty and provided residents opportunities to develop as teachers. While we collected no data analyzing the effectiveness of our residents as teachers, their commitment likely contributed to the success of the course. In our review of the literature, few mentioned resident contributions to physical exam courses. In one study, chief residents helped lead physical exam rounds ( Fagan *et al.,* 2003)while in another study residents developed a physical exam course outline for co-residents with didactic support from faculty and fellows ( Wijeratne *et al.,* 2018). Based on the preliminary findings in our study there may be further opportunities to expand resident-driven physical exam courses to other institutions.

There were several limitations to this study. We conducted our course at only one institution with a small group of students who self-selected to take the elective. Results were thus difficult to generalize given the small sample size and selection bias by motivated students. Furthermore, the course was only two weeks long without follow up to assess retention. Despite this flaw, there is some evidence to support that even brief interventions of intensive physical exam training can have durable retention of knowledge for at least four-to-12 months ( Perrig *et al.,* 2016). Such findings suggest our students developed skills that will persist well into their internships where further refinement can take place. Due to logistical constraints, we were unable to create a pre-course OSCE or multimedia assessment. Future studies would benefit from a more comprehensive pre-course and post-course testing to ascertain knowledge acquisition. Additionally, our study did not have a control group of students to prove the efficacy of our intervention compared to a baseline. Lastly, residents and faculty underwent no formal training, possibly creating differences in teaching quality from year to year and student to student.

## Conclusion

In conclusion, students demonstrated improved knowledge, skill, and comfort with the physical examination after a two-week elective. The course was innovative and comprehensive in its incorporation of evidence-based medicine, clinical decision making, visual literacy, POCUS, and residents as teachers. Further research is needed to study the optimal physical exam course for retention of knowledge and impact on patient care.

## Take Home Messages

• We created a two-week elective course comprised of resident and faculty-led didactics, student-led journal discussions, bedside ultrasound, and physical exam rounds.
• We included additional activities on biostatistics, cognitive biases, visual literacy, and medical decisionmaking.
• Twentytwo fourth year medical students demonstrated statistically significant improvement on multiple choice pre-course and post-course assessments (56.8% vs 77.1%, p < 0.001).
• 95.5% of students reported feeling “confident” using the physical exam by the end of the course.
• Resident contributed roughly half of all teaching hours to alleviate faculty teaching hours and facilitate learn.

## Notes On Contributors

Ryan Dean, MD was an Internal-Medicine Pediatrics resident at Prisma Health-Upstate. He was also a resident instructor at the Unviersity of South Carolina School of Medicine - Greenville. Dr. Dean developed the physical exam course curriculum, facilitated the course, processed data, and wrote the manuscript. ORCiD: https://orcid.org/0000-0001-6773-658X

Dustin Morrow, MD is chief of the Division of Emergency Ultrasound within the Department of Emergency Medicine at Prisma Health-Upstate. He is Director of Ultrasound Education and faculty at the University of South Carolina School of Medicine - Greenville. Dr. Morrow participated in curriculum development, taught the course, and reviewed the manuscript.

Steven Connelly, MD is hospitalist within the Department of Internal Medicine at Prisma Health-Upstate. He is also a clinical professor at the University of South Carolina School of Medicine - Greenville. Dr. Connelly oversaw the physical exam course, developed curriculum, taught the course, and reviewed the manuscript.
