# Supplementary material for: A Multifaceted Resident-Led Physical Exam Course
Source: MedEdPublish (2016). 2021 May 3;10:109. [Version 1] doi: 10.15694/mep.2021.000109.1 (PMC10939526; doi:10.15694/mep.2021.000109.1)
Supplement: Peer review history

# Reviewer report: Reviewer response for version 1

**DOI:** 10.21956/mep.18951.r27119

**Published:** 2021-10-12

**Authors:** Balakrishnan Nair

**Affiliations:** 1 Centre for Medical Professional Development and University of Newcastle

This review has been migrated. The reviewer awarded 3 stars out of 5 Focused and problem-oriented physical examination is important in diagnosis. Physical examination is a way of “connecting “with patients too. Often this is a neglected area in medical education. So this article to revive evidence-based physical examination is to be applauded. However, the project had only 33 students over 2 years and the training for each group lasted only two weeks. The assessment of the outcome should be the accuracy of the physical examination. I assume “POCUS “ is the point of care ultrasound. A larger sample size and longer training will be a good follow up for this important project

Reviewer Expertise:

NA

No decision status is available

# Reviewer report: Reviewer response for version 1

**DOI:** 10.21956/mep.18951.r27121

**Published:** 2021-10-12

**Authors:** Ken Masters

**Affiliations:** 1 Sultan Qaboos University

This review has been migrated. The reviewer awarded 3 stars out of 5 The article deals with a multifaceted resident-led physical exam course. Overall, the context has been well-introduced, and the course reasonably well-described, and the study well-conducted.Given the small sample size, the problem that the authors would wish to overcome is the broader applicability. Although the small sample size is mentioned in the paper as a limitation, it is still a problem as far as the value to outside readers goes. A starting point to overcoming this would be to supply a supplementary file that gives further details of the course. This would allow readers to examine the course in greater depth, and be able to apply it to their own situations, with suitable adjustments.Similarly, rather than simply refer to “follow-up surveys”, the value of the paper would have been increased if the authors had supplied a copy of the questionnaire and given details of these surveys, how conducted, etc. On the course, there were 22 students, but 33 people (faculty, chief residents, and residents) involved in the instruction. Perhaps the authors could comment on the sustainability of running such a course with high numbers of students (again, a more detailed break-down of the course structure might have given an indication of the time involved per instructor). Related to this, the “goal” ratio, but not the actual ratio is given. It would be useful to know the actual ratio.So, the paper is an interesting read, but the authors could have done a little more to increase its value to the reader.

Reviewer Expertise:

NA

No decision status is available

# Reviewer report: Reviewer response for version 1

**DOI:** 10.21956/mep.18951.r27120

**Published:** 2021-05-03

**Authors:** P Ravi Shankar

**Affiliations:** 1 American International Medical University

This review has been migrated. The reviewer awarded 4 stars out of 5 This is an interesting and well-written manuscript. The elective on physical examination has been well described. The authors have also highlighted the limitations of their study. One of my queries is why did so few medical students take this course? Only twenty-two students participated. What is the annual student intake at the school? Did the same residents and faculty facilitate the course from 2017 to 2019?

Reviewer Expertise:

NA

No decision status is available
